# Influence of the Chemical Structure of Perylene Derivatives on the Performance of Honey-Gated Organic Field-Effect Transistors (HGOFETs) and Their Application in UV Light Detection

**DOI:** 10.1021/acsaelm.4c01773

**Published:** 2024-11-22

**Authors:** Jose Diego Fernandes Dias, Douglas Henrique Vieira, Theodoros Serghiou, Carlos J. Rivas, Carlos J. L. Constantino, Liliana B. Jimenez, Neri Alves, Jeff Kettle

**Affiliations:** † Department of Physics, School of Sciences and Technology, São Paulo State University (UNESP), Presidente Prudente 15385-000, São Paulo, Brazil; ‡ James Watt School of Engineering, University of Glasgow, G12 8QQ Glasgow, Scotland, U.K.; § Departamento de Química Orgánica, Facultad de Ciencias Químicas, Universidad Nacional de Córdoba. INFIQC, Instituto de Investigaciones en Físicoquímica de Córdoba (CONICET-UNC). Córdoba X5000HUA, Argentina

**Keywords:** HGOFETs, sustainability, chemical structure, perylenes, UV light detector

## Abstract

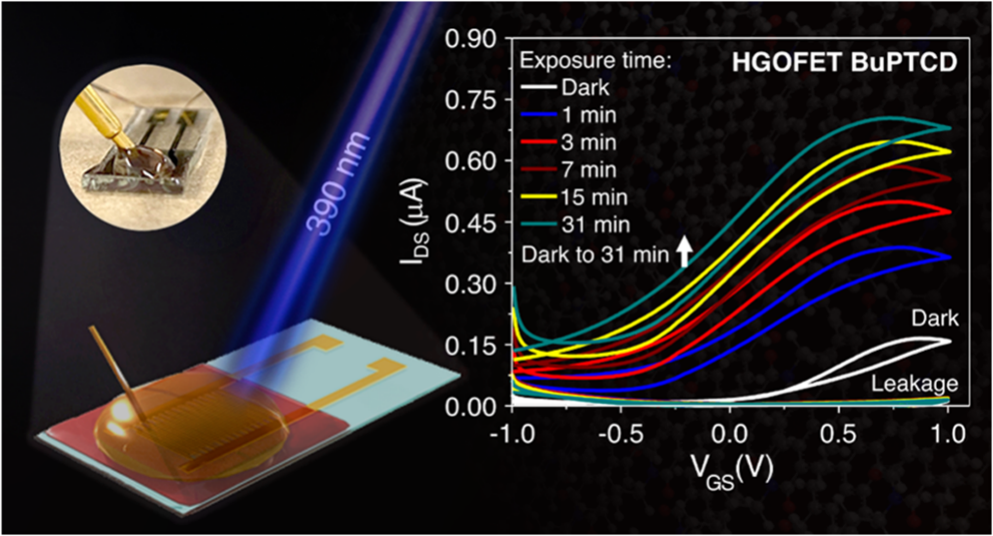

Electronics based on natural or degradable materials are a key requirement for next-generation devices, where sustainability, biodegradability, and resource efficiency are essential. In this context, optimizing the molecular chemical structure of organic semiconductor compounds (OSCs) used as active layers is crucial for enhancing the efficiency of these devices, making them competitive with conventional electronics. In this work, honey-gated organic field-effect transistors (HGOFETs) were fabricated using four different perylene derivative films as OSCs, and the impact of the chemical structure of these perylene derivatives on the performance of HGOFETs was investigated. HGOFETs were fabricated using naturally occurring or low-impact materials in an effort to produce sustainable systems that degrade into benign end products at the end of their life. It is shown that the second chain of four carbons at the imide position present in perylenes *N*,*N*′-bis(5-nonyl)-perylene-3,4,9,10-bis(dicarboximide) (PDI) and *N*,*N*′-bis(5-nonyl)-1-naphthoxyperylene-3,4,9,10-bis(dicarboximide) (PDI-ONaph) reduces π-stacking interaction in the active layer, leading to lower AC conductivity and the non-functionality of HGOFETs. On the other side, the chain-on molecular orientation in the film of *N*,*N*′-dibutylperylen-3,4:9,10-bis(dicarboximide) (BuPTCD) was fundamental for the efficiency of HGOFETs, showing a better performance than the HGOFETs of *N*,*N*′-bis(2-phenylethyl)-3,4:9,10-bis(dicarboximide) (PhPTCD), which has a face-on molecular orientation. Finally, the HGOFETs of BuPTCD and PhPTCD are good candidates as UV light detectors and are used for the detection of UV radiation.

## 1 Introduction

Due to the growth of consumer electronics over the past 20 years, the importance of sustainability in electronics has come to the fore, particularly because of large volumes of electronic waste (e-waste) and the need to decarbonize many industries.^1^ As a result, there is substantial work on the development of electronic devices made with “sustainable” materials, which are based on either bioderived, biodegradable, organic, or biocompatible materials.^2−4^ Manufacturing devices with such materials is desirable, as it allows for devices, circuits, and systems to be decomposed into non-hazardous end products after the lifespan of devices, reducing the impact of waste in the environment. Transistors are a key component and are used in nearly all modern applications, including computation, data storage, power supplies, sensing, and communication. Nevertheless, due to their multilayered structure, transistors are relatively complex devices and require different classes of materials (i.e., conductors, dielectrics, semiconductors) for different elements within devices.^5^

Organic semiconductor compounds (OSCs) are materials with inherent properties of low-temperature processability, low weight, and soft nature, which enable this technology to be used for high-throughput, large-area electronics. They provide an alternative approach for manufacture and will help the information and communication technology (ICT) industry to avoid depletion of non-renewable or finite resources and could reduce the industry’s carbon footprint as a result of the lower energy consumption involved in their processing into thin films, as well as energy embodied in the material manufacture.^6,7^ Among these OSCs, perylene derivatives stand out due to their optical properties (absorption and emission), chemical/thermal stability, biocompatibility, and the possibility of making controlled modifications in their chemical structure to adjust both optical and electrical properties according to specific application requirements.^8−11^ Perylene materials were chosen, as they can be easily synthesized using simple organic reactions with no need for high-impact transition metals as catalysts, yielding high synthetic efficiencies. Substitutions at the “peri” positions primarily affect solubility and aggregation, while modifications at the “bay” and “ortho” positions efficiently tune optical and redox properties. Regarding sustainability, adding non-polar groups at the “peri” positions reduces solubility in water and lowers volatility, minimizing the environmental dispersion of these materials and enhancing their sustainability.^12^ This is the case of PDIs (*N*,*N*′-dibutylperylen-3,4:9,10-bis(dicarboximide) (BuPTCD), *N*,*N*′-bis(2-phenylethyl)-3,4:9,10-bis(dicarboximide) (PhPTCD), *N*,*N*′-bis(5-nonyl)-perylene-3,4,9,10-bis(dicarboximide) (PDI), *N*,*N*′-bis(5-nonyl)-1-naphthoxyperylene-3,4,9,10-bis(dicarboximide) (PDI-ONaph)) presented in this work.

For practical applications, devices with low-voltage (i.e., *V*_DS_ < *V*) operation are essential, and honey-gated organic field-effect transistors (HGOFETs) have emerged as potential devices owing to their ease of fabrication and low operating voltages by utilizing an electrolyte instead of a traditional gate dielectric. The use of honey as an electrolyte in devices can present potential challenges, particularly due to its variable composition, which can fluctuate based on factors such as floral origin, climatic conditions (including humidity and temperature),^13^ and the maturation time of the honeycomb. However, honey offers significant advantages that make it an attractive option for electronic applications. It is naturally occurring, abundant, and naturally biodegradable, and its ease of processing provides a simple and effective alternative in contexts where sustainability and cost-effectiveness are key considerations. Also, it has been reported as a rapid and simple method to evaluate a semiconductor’s quality and its charge transport characteristics,^14,15^ making it a more accessible choice in this way, since ion gels require several preparation steps in a controlled atmospheric environment, adding complexity. The fabrication process of HGOFETs is simple, involving only the manual deposition of an electrolyte under the OSC film (top-gate), which covers the entire channel, and the addition of the gate electrode. By applying an electrical potential to the gate, an electrical double layer (EDL) is formed at the electrolyte/OSC interface, and in this way, the current in the channel is controlled. EDLs achieve high capacitance (*C* ≈ 1 μF cm^–2^) and enable EGOFETs to operate at low voltages (*V*_DS_ < 1 V).^16−18^ HGOFETs are compatible with applications that mimic biological processes, are versatile for use as sensors, and have the potential to serve as tools for researching chemical and physical phenomena.^19−22^ Considering the latter, small changes in the OSC chemical structure can alter the supramolecular arrangement in the active layer^23,24^ and, consequently, the properties and efficiency of electronic devices.^25^ Therefore, establishing a relationship among the chemical structure, active layer, and electronic device performance is crucial for the advancement and improvement of organic electronic devices.

**Figure 1:**
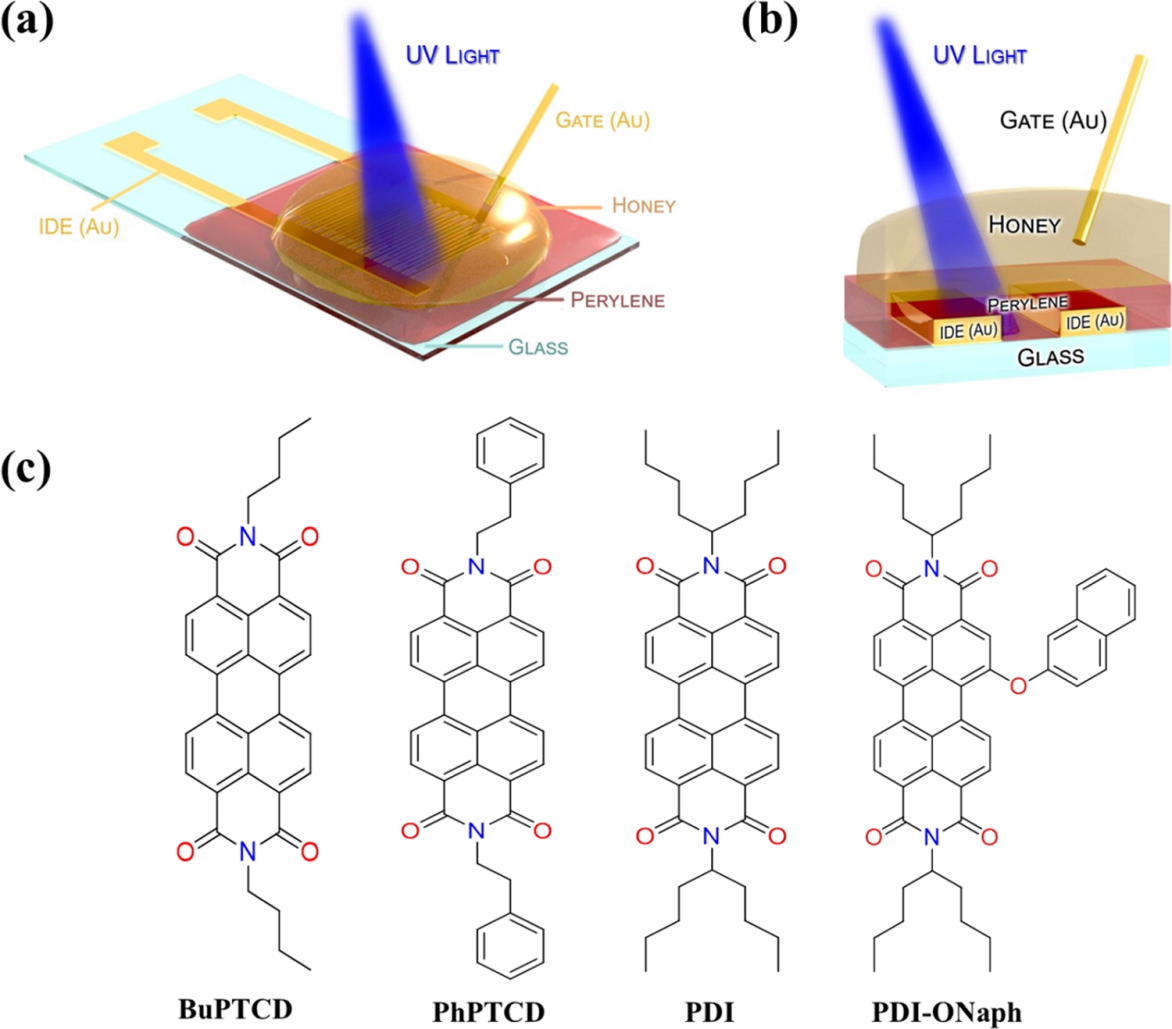
(a) Illustration of HGOFETs using honey as electrolytic dielectric layers, (b) illustration of the HGOFET cross section, and (c) chemical structures of BuPTCD, PhPTCD, PDI, and PDI-ONaph.

In this paper, we demonstrate the performance of the HGOFETs based on benign materials, meaning that the products at the end of life do not have an impact on the environment. The HGOFETs were fabricated utilizing honey as the electrolyte, perylene as the active layer, gold for the contacts (source, drain, and gate), and glass as the substrate (Figure 1a,b). Honey, a dielectric material, presents various advantages, namely, no environmental and health hazards, low cost, wide accessibility, edible substance, long shelf life, and biocompatibility.^3^ Perylene is a biocompatible organic semiconductor, and gold (Au) is often used in medical applications in vivo.^26,27^ We elucidate the influence of the chemical structure of perylene derivatives on the electrical properties of the HGOFET’s active layer. To achieve this, films of BuPTCD, PhPTCD, PDI, and PDI-ONaph (chemical structure in Figure 1c) were deposited onto interdigitated Au electrodes and characterized through impedance, absorption, and emission spectroscopy. Through the analysis of the performances, it is demonstrated that the addition of a second chain of four carbons at the imide position reduces π-stacking interaction in the active layer, rendering the fabrication of HGOFETs unfeasible. Then, we underscore the pivotal role of molecular orientation in ensuring that BuPTCD HGOFETs outperform PhPTCD HGOFETs. Finally, we showcase that BuPTCD and PhPTCD HGOFETs present promising alternatives for the development of UV light detectors.

## 2 Materials and Methods

### 2.1 Materials

BuPTCD (MW = 502.56 g mol^–1^) and PhPTCD (MW = 602.15 g mol^–1^) were synthesized by J. Duff of Xerox Resource Centre, Canada, and provided by Professor R. F. Aroca of Windsor University, Canada. The PDI (MW = 642.84 g mol^–1^) and PDI-ONaph (MW = 785.00 g mol^–1^) were synthesized following the procedure previously reported.^28^ The organic solvents trifluoroacetic acid (TFA, C_2_HF_3_O_2_) and dichloromethane (DCM, CH_2_Cl_2_) were of high purity grade (≥99%) and were purchased from Sigma-Aldrich (St. Louis, MO, USA). Both organic solvents were used to prepare 1 × 10^–6^ mol L^–1^ solutions of perylenes dissolved in a mixture of DCM/TFA at a 9:1 (v/v) ratio. The electrolytic dielectric layer was liquid, ca. 10 μL of honey dripped onto perylene films, aligned with the source, and drain electrodes. The honey utilized in this experiment was purchased from a local market (commercial honey produced by Rowse Light & Mild Honey packed in the United Kingdom).

### 2.2 Fabrication of Films and HGOFETs

The interdigitated electrodes (IDEs) used for the source and drain were patterned on a glass substrate by photolithography and consisted of 20 nm of chrome (Cr) followed by 100 nm of gold (Au). Each finger within the IDEs has dimensions of 3 mm length (*L*), and 10 μm width (*W*), 10 μm spacing from one finger to the other for a total of 50 pairs of digits (100 fingers). The perylene derivative thin films were deposited over the IDEs by physical vapor deposition (PVD). All perylene derivatives were evaporated to a rate between 0.1 and 0.2 nm s^–1^, monitored in situ by a quartz crystal microbalance to a thickness of 100 nm, which was calibrated with a profilometer. A 10 μL portion of honey was used as a liquid electrolytic dielectric layer deposited by drop casting onto perylene films aligned to the center of the IDE area. To complete the transistor fabrication, a gold tip (gate electrode) was immersed into the honey droplet, resulting in transistors with a top-gate, bottom-contact architecture.

### 2.3 Characterization

The transfer and output curves of the HGOFETs were carried out using a semiconductor characterization system (Keithley 4200 SCS). The impedance spectroscopy of perylene derivative films was performed using a Solartron impedance analyzer with a 1296 dielectric interface system, using an AC signal of 100 mV, DC levels of 0 and 1 V, and a frequency range from 0.1 Hz to 1 MHz. The film morphology and thickness of the different perylene derivatives deposited on glass plates was characterized at a nanometer scale by AFM images recorded using a Bruker dimension icon AFM in tapping mode. The topographic images were analyzed using NanoScope analysis 3.0. UV–vis absorption measurements were performed on the perylene derivative films deposited on glass substrates using a UV-2600i Shimadzu spectrophotometer, covering the wavelength range from 350 to 700 nm. Fluorescence measurements were conducted utilizing an Edinburgh Instruments FS5 spectrofluorometer with an excitation wavelength set at 514 nm. X-ray diffraction was carried out in a Bruker D8 Venture kappa geometry diffractometer equipped with a Photon-II CPAD detector and dual (Cu and Mo) ImS 3.0 microfocus sources. The characterization was performed in the dark and under UV radiation using an Ansmann 1600-0307 LED Rechargeable Work Light, 5 W, 3.7 V, as the 390 nm source, calibrated with a PM100D—Compact Power and Energy Meter Console, Digital 4″ LCD.

## 3 Results and Discussion

**Figure 2:**
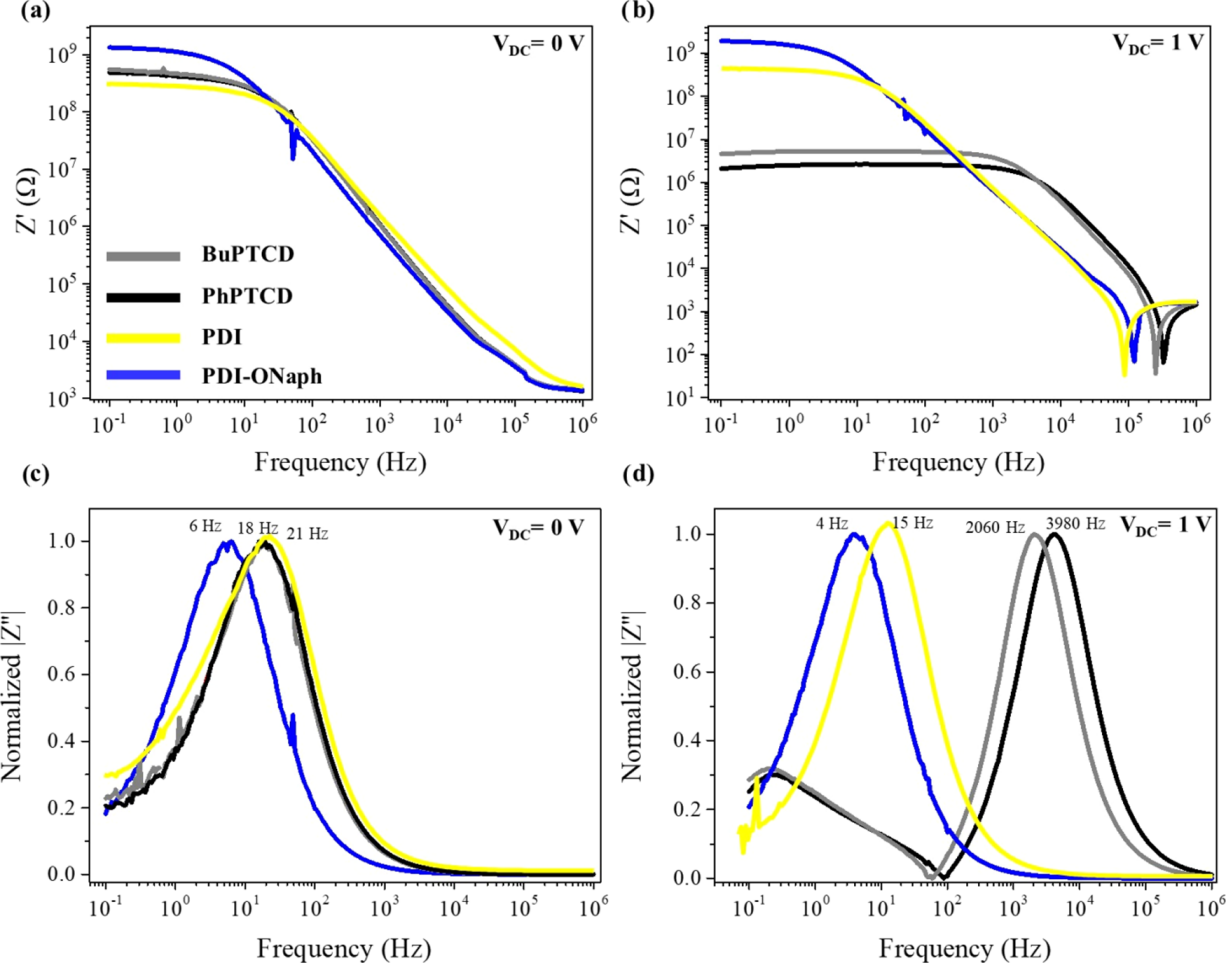
(a, b) *Z’* versus *f* and (c, d) *|Z’’|* versus *f* graphs in the dark are illustrated for BuPTCD (gray line), PhPTCD (dark line), PDI (yellow line), and PDI-ONaph (blue line) under direct current conditions of 0 and 1 V, respectively.

To initiate this work, the four perylene derivative films were deposited onto interdigitated gold electrodes, and their electrical properties were investigated through impedance measurements, aiming to comprehend their characteristics before application in a transistor. Figure 2a shows the real impedance versus frequency (*Z’ – f*) curves of Au/perylene-derivative/Au devices under dark conditions, acquired by applying a constant voltage (*V*_DC_) of 0 V and an alternating voltage (*V*_AC_) of 100 mV. When *V*_DC_ is set to 0 V, the *Z’–f* curves for perylene derivative films exhibit a similar shape. The impedance profile indicates a Maxwell–Wagner relaxation, modeled by a parallel RC circuit,^29^ characterized by a high impedance plateau (∼10^9^ Ω) at low frequencies (*f* ≤ 100 Hz), a region in which impedance is predominantly governed by the resistance of the semiconductor. As the frequency increases, the capacitive impedance becomes dominant (*Z*_capacitor_ = 1/*j*ω*C*). This behavior is typical of semiconductors where charge transport is limited by the mobility of charge carriers within the semiconductor as well as by the interface between the electrodes and the organic material. Consequently, the transition from resistive to capacitive behavior occurs because the carriers cannot follow the alternating electric field beyond a certain operational frequency. However, upon voltage increasing to *V*_DC_ = 1 V, the impedance of BuPTCD and PhPTCD films decreases to around 10^6^ Ω (*f* ≤ 100 Hz), while the impedance for PDI and PDI-ONaph films remains unchanged (Figure 2b). At high frequencies (*f* ≥ 10^5^ Hz), films of perylene derivatives exhibit nearly identical voltage-independent impedance. Additionally, minimal spikes are observed across all perylene derivative films (Figure 2b). This resembles the minimal spikes observed in the impedance of RLC circuits and is attributed to the transition from the capacitive to the inductive behavior.^30,31^ A similar phenomenon can also be found when there are negative capacitance effects in semiconductors.^32^ However, investigating the detailed origin of these minimal spikes in the perylene derivative films spectrum goes beyond the scope of this work. The analysis of these results in terms of normalized imaginary impedance modulus versus frequency (*|Z’’| – f)* plots, as shown in Figure 2c,d, respectively, reveals that for *V*_DC_ = 1 V, there is a shift of the dielectric relaxation peak (*f*_max_) toward higher frequencies in BuPTCD and PhPTCD films compared to *V*_DC_ = 0 V. However, no significant alteration is observed in the spectra of PDI and PDI-ONaph films. The shift of *f*_max_ to higher frequencies can also be observed under light exposure^33^ and elevated temperatures,^34^ both of which were attributed to an increase in the number of carriers in the active layer. Therefore, the results suggest an increase in the number of carriers for BuPTCD and PhPTCD films under a bias of *V*_DC_ = 1 V. In other words, this voltage is sufficient for efficient charge injection into the device channel, indicating that contact does not limit the conduction. Meanwhile, for PDI and PDI-ONaph films, the electric current does not exhibit efficient injection, as their properties remain unchanged. This indicates that both films (PDI and PDI-ONaph) display conduction that may be limited by the interface if the lack of change is due to contact barriers at the Au/perylene interface or limited by volume, in case the lack of change is attributed to the difficulty of current passage between molecules. The different observed properties can be attributed to the chemical structure, i.e., the influence of different branches on electrical transport, which will be further discussed. It is important to highlight that when shifted, the BuPTCD and PhPTCD spectra reveal a second imaginary impedance peak at low frequencies, which can be associated with parasitic current at electrodes or interface states.

AC conductivity was evaluated from impedance data using eq 1,^35^ where *k* is the cell constant, obtained using the method proposed by Olthuis et al.^36^
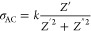
1

**Figure 3:**
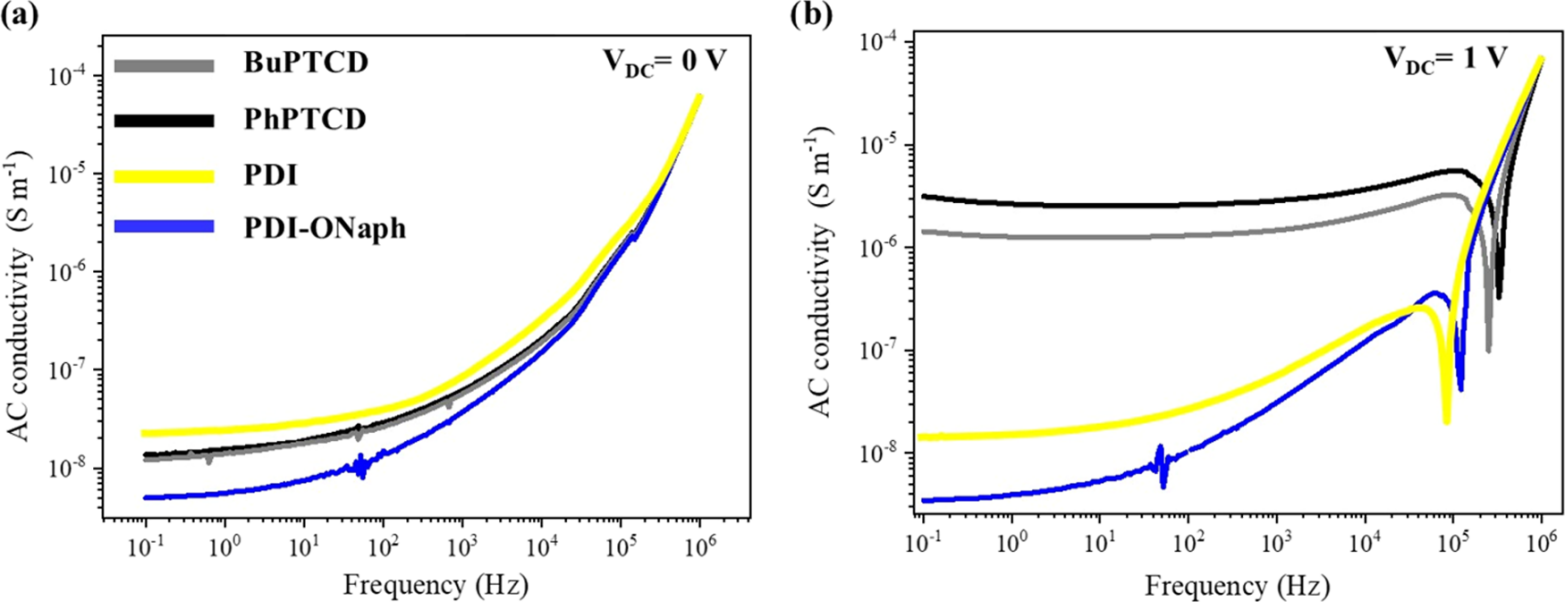
σ_AC_–*f* curves for BuPTCD (gray line), PhPTCD (black line), PDI (yellow line), and PDI-ONaph (blue line) under (a) *V*_DC_ = 0 V and (b) *V*_DC_ = 1 V.

The AC conductivity versus frequency (σ_AC_–*f*) plots of Au/perylene-derivative/Au devices at a bias of *V*_DC_ = 0 V are presented in Figure 3a. The perylene derivative films exhibit slightly different behaviors at low frequencies (*f <* 10^2^ Hz). However, at *V*_DC_ = 1 V (Figure 3b), the AC conductivity significantly changes for the BuPTCD and PhPTCD films. The AC conductivity of BuPTCD and PhPTCD films increases over the range of 1 × 10^–^–1 × 10^5^ Hz, achieving an increase of approximately 1 × 10^2^ S m^–1^ for *f <* 1 × 10^2^ Hz. This increase in AC conductivity is attributed to the enhanced injection of charge carriers “*N*” (). On the other hand, the AC conductivity of PDI and PDI-NOnah films remains practically unchanged. There are minimal spikes at high frequencies in the AC conductivity for all perylene derivative films at *V*_DC_ = 1 V. These spikes are a consequence of the impedance spikes shown in Figure 2b and of eq 1 used to determine the AC conductivity. The difference in the behavior of BuPTCD and PhPTCD films compared to PDI and PDI-ONaph films at *V*_DC_ = 1 V was investigated through the absorption and emission spectrum. The electrical conductivity of organic films with π-conjugation is dependent on intermolecular distance, carrier mobility, and charge carrier concentration.^37^ Upon analyzing the absorption spectra of perylene derivative films presented in Figure S1 in the Supporting information and comparing them with their respective solutions at low concentrations (monomeric state), it is noteworthy that a broadening of the absorption band (both blue and red shifts) and changes in the relative intensity between vibrational transitions (0–0, 0–1, and 0–2) are observed in the absorption spectra of both BuPTCD and PhPTCD films. This phenomenon is attributed to intermolecular interaction and the formation of molecular aggregates, requiring small intermolecular distances and effective π-stacking interaction, suggesting the formation of compact films.^38^ However, in the case of absorption spectra for PDI and PDI-ONaph films, no significant broadening of the absorption band (either blue or red shift) or abrupt changes in relative intensities attributed to vibrational transitions were observed (Figure S1in the SI), suggesting that larger intermolecular distances are present, leading to weaker π-stacking interaction. The emission spectrum of perylene derivatives (Figure S2 in the SI) shows that the BuPTCD and PhPTCD films exhibit maximum emission with a more pronounced red shift compared with PDI and PDI-ONaph films. As mentioned in references,^39,40^ films with closer-packed molecules tend to increase the π-stacking interaction, which can result in a decrease in the energy gap between the first excited electronic state (S_1_) and the ground state (S_0_), consequently leading to a red shift in the emission spectrum. Therefore, the emission spectrum results also suggest a stronger π-stacking interaction in the BuPTCD and PhPTCD films compared to the PDI and PDI-ONaph films, consistent with the absorption results obtained. Analyzing the differences in the chemical structures of perylene derivatives with higher packing (BuPTCD and PhPTCD) and those with lower packing (PDI and PDI-ONaph), it becomes evident that the presence of a second chain with four carbon atoms in the imide position is responsible for the intermolecular separation. This is the principal difference between BuPTCD and PDI. Finally, we can conclude that the smaller intermolecular distances in BuPTCD and PhPTCD films may facilitate the movement and injection of carriers into the active layer when under bias at *V*_DC_ = 1 V.

**Figure 4:**
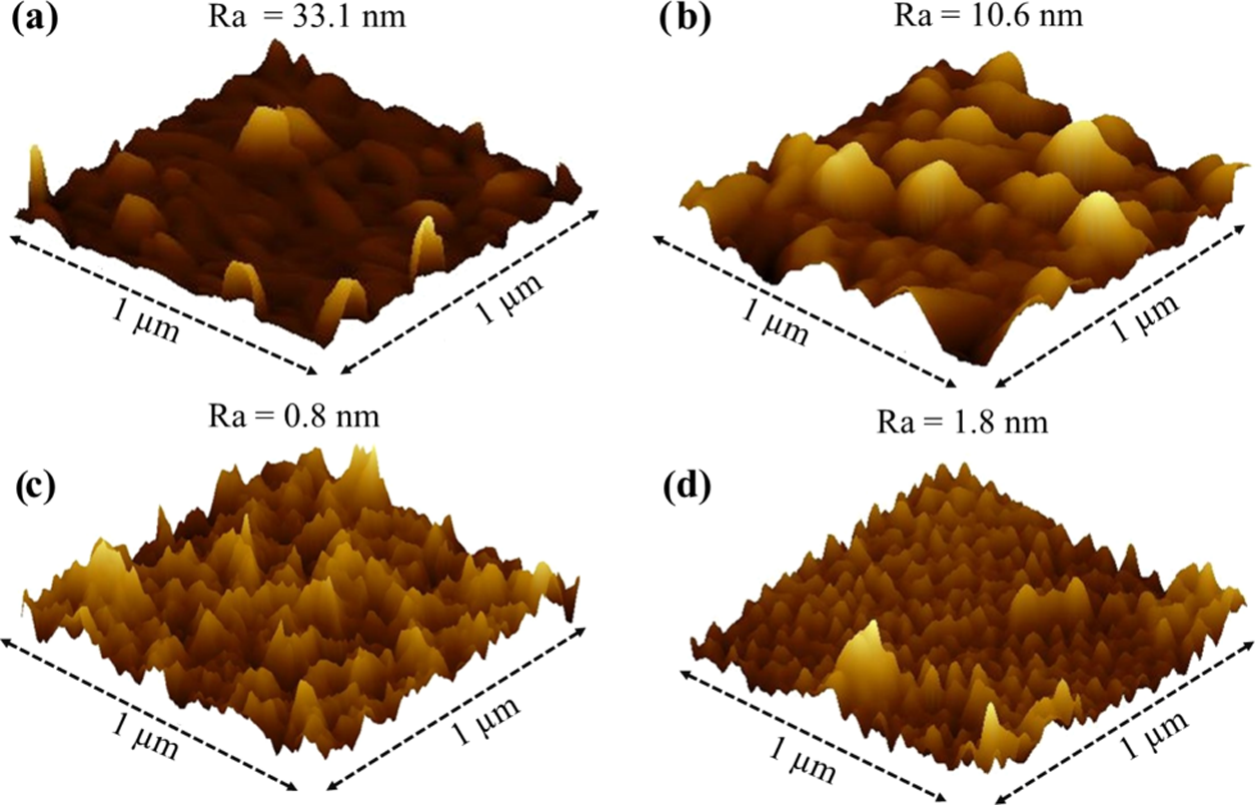
AFM images of (a) BuPTCD, (b) PhPTCD, (c) PDI, and (d) PDI-ONaph PVD films with a size 1 μm × 1 μm and their respective roughness averages.

Figure 4 presents the AFM images of perylene films acquired with a size of 1 μm × 1 μm, along with their respective roughness averages (Ra). BuPTCD and PhPTCD films, as shown in Figure 4a,b, respectively, exhibit surfaces with large aggregates and higher roughness averages compared to those observed in PDI and PDI-ONaph films (Figure 4c,d respectively). As indicated by absorption (Figure S1) and emission (Figure S2) spectra, BuPTCD and PhPTCD films have higher packing than PDI and PDI-ONaph films. Therefore, the AFM results suggest a correlation where molecules with stronger π-stacking interactions form tightly packed films, exhibiting greater roughness and larger aggregates. However, molecules with weaker π-stacking interactions produce less packed films characterized by smoother surfaces and smaller aggregates.

**Figure 5:**
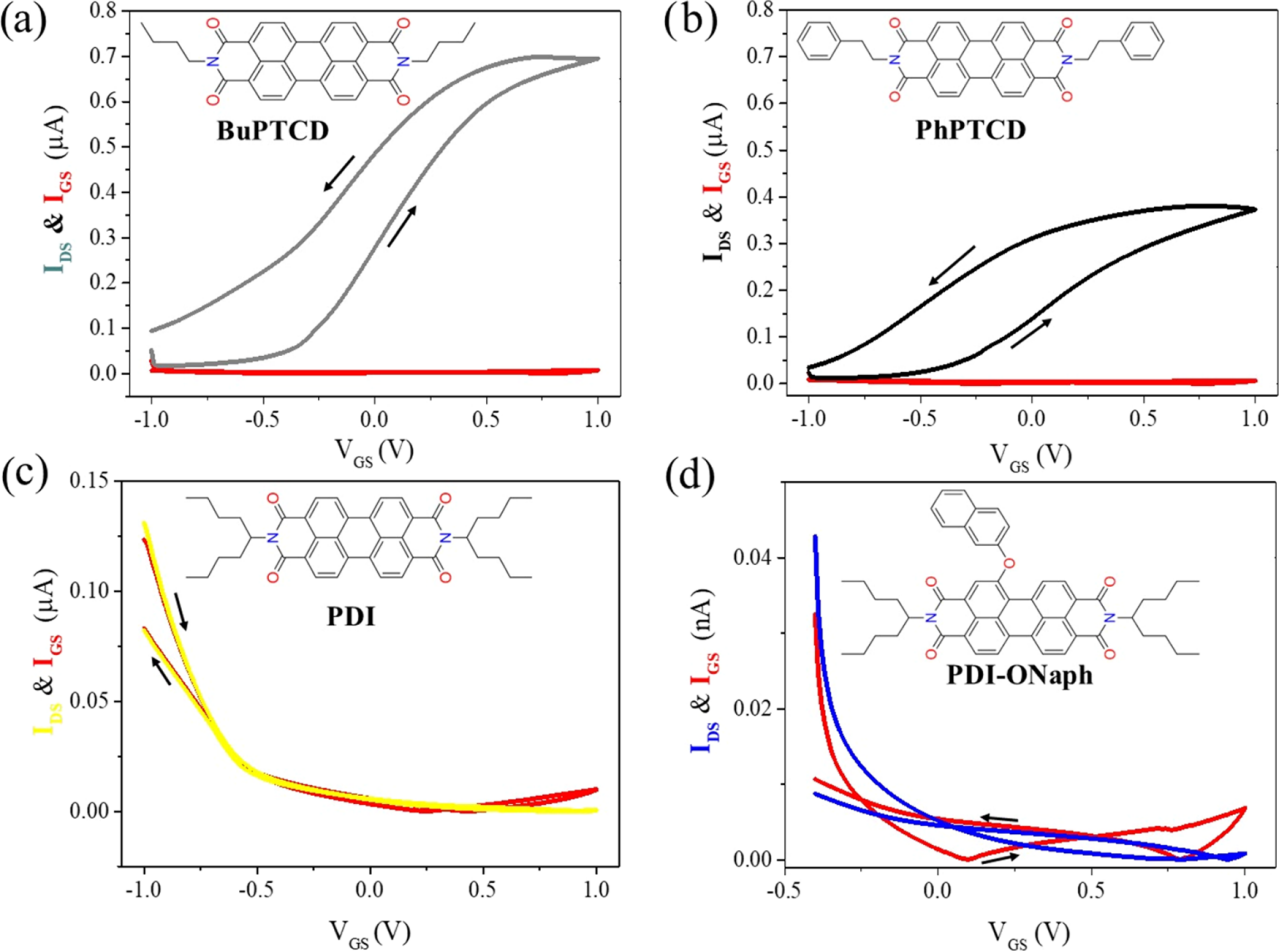
Transfer curves of HGOFETs for sample 1 of (a) BuPTCD, (b) PhPTCD, (c) PDI, and (d) PDI-ONaph, measured under *V*_DS_ = 1 V. The red lines represent the corresponding leakage currents.

The transistor architecture was completed by depositing honey as an electrolyte over the perylene derivative films and connecting a gate electrode to it (as illustrated in Figure 1a). Figure 5 shows the double sweep transfer (*I*_DS_–*V*_GS_) characteristics of the perylene-based HGOFETs, along with their respective leakage current (*I*_GS_–*V*_GS_) at a scanning rate of 10 mV s^–1^ and *V*_DS_ = 1 V. Due to the substantial water content in honey, we limited our gate voltage testing to the range of −1 to 1 V to avoid initiating the electrolysis of water, potentially altering the properties of the honey and affecting the device’s performance. Figure 5a,b reveals that HGOFETs fabricated with BuPTCD and PhPTCD, respectively, exhibit *I*_DS_ modulation by the gate voltage with low *I*_GS_, confirming their operation as transistors. Both devices also presented large anticlockwise hysteresis. Conversely, Figure 5c,d demonstrates that *I*_DS_ and *I*_GS_ are in the same magnitude for HGOFETs fabricated with PDI and PDI-ONaph, indicating non-functional transistors. The non-working of the transistor with PDI and PDI-ONaph may be associated with the effect discussed in the data from Figure S1, namely, the increased intermolecular distance caused by the chemical structure hindering current flow in the channel. This is also consistent with the results observed in the impedance analysis (Figure 2).

Transfer curves were measured in three distinct BuPTCD and PhPTCD HGOFETs to obtain the values of the performance parameters. Transfer data were used to determine the transistor’s performance parameters; *I*_on_/*I*_off_ was determined using the maximum and minimum current observed in the transfer, and the on-current-to-leakage ratio (*I*_DS_/*I*_GS_) was evaluated to make sure that the current was properly modulated by the gate electric field. The threshold voltage (*V*_TH_) was obtained from an *x*-axis intercept in the √*I*_DS_–*V*_GS_ curve, and the mobility-capacitance product (μ_s_*C*_i_) was determined from the slope of this same plot by utilizing eq 2.
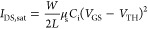
2This is applicable at (|*V*_DS_| > |*V*_GS_*– V*_TH_|).^41^ The transconductance (*g*_m_) and subthreshold swing (SS) were also obtained using the following equations,^42,43^ where *V*_DS_ is a value constant (const.)
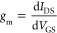
3
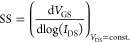
4

**Figure 6:**
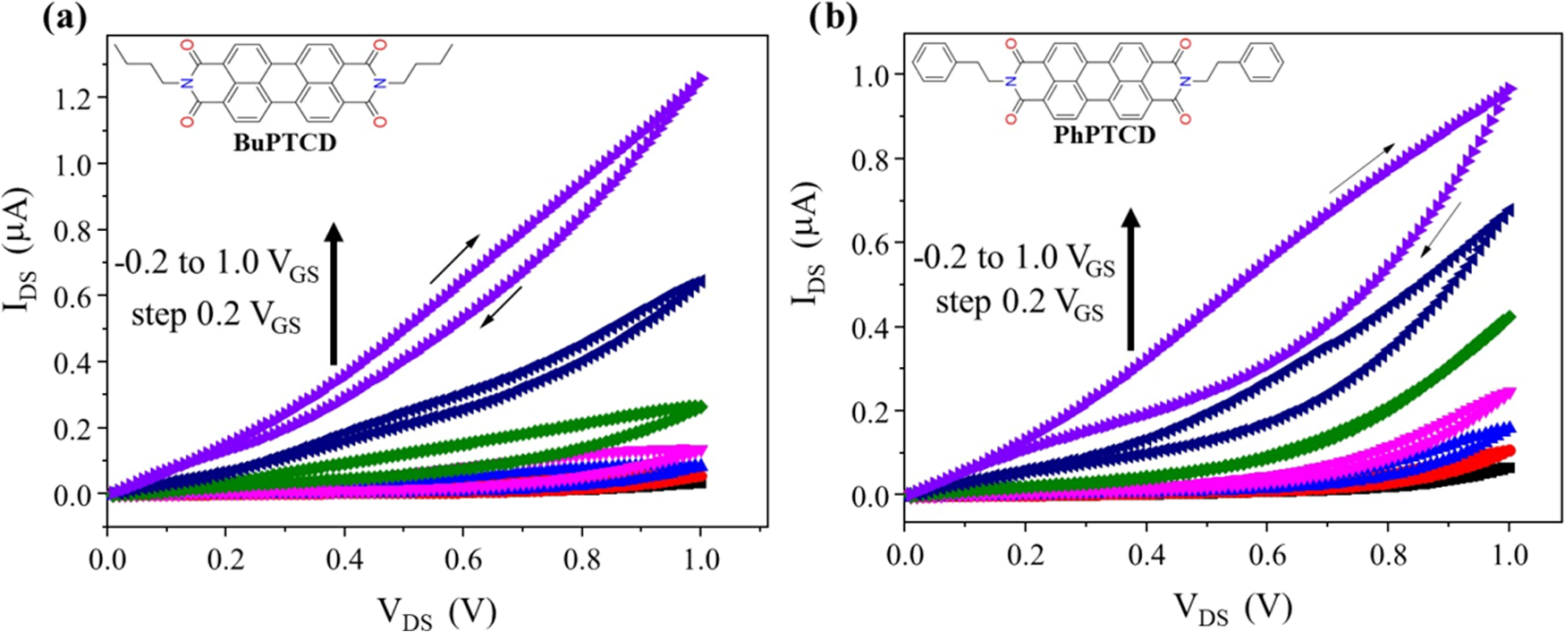
Output curves (*I*_DS_–*V*_DS_) with *V*_GS_ ranging from −0.2 to 1 V in a 0.2 V increase and *V*_DS_ ranging from 0 to 1 V for (a) BuPTCD and (b) PhPTCD.

It is important to emphasize that due to the output curve not reaching the saturation regime (Figure 6), artifacts are present, leading the calculated parameters to reflect the device’s performance rather than the intrinsic properties of the material.

Table 1 displays the performance parameters obtained from the transfer curves of BuPTCD and PhPTCD HGOFETs. The mobility-capacitance product (μ_s_*C*_i_) is a crucial parameter for electrolyte-gated transistors,^18,41,44^ especially in situations where it is difficult to determine a nominal value for capacitance, for example when the capacitance is voltage-dependent.^45^ Here, we observe that the values of μ_s_*C*_i_ are higher for BuPTCD than those for PhPTCD HGOFETs. Since honey capacitance is expected to be the same for both films, this suggests that the values of μ_s_ are greater for the BuPTCD HGOFETs. These results indicate that charge carriers move more easily in BuPTCD HGOFETs than in PhPTCD. In addition, when analyzing the channel current (*I*_DS_) through the transfer curves (Figures 5a,b and S3a–d, SI), it is evident that the *I*_DS_ values for BuPTCD HGOFETs are higher than those for PhPTCD. These results align with expectations when analyzing the molecular orientation within both films. It is noted from the literature that the charge carrier mobility of molecules with π-conjugation is higher in the π-stacking direction.^25^ The molecular orientation in the BuPTCD film is chain-on, with the chromophore plane perpendicular to the substrate surface and supported on its smaller base.^46^ Therefore, the π-stacking interactions occur horizontally and parallel to the substrate surface (Figure S4a). In contrast, the molecular orientation in the PhPTCD film is face-on, with the chromophore plane parallel to the substrate surface.^47^ Moreover, its π-stacking direction is vertical, perpendicular to the substrate surface (Figure S4b). Thus, for a device with a top-gate, bottom-contact architecture (Figure 1a), where charge carriers are directed to preferentially move horizontally (in the direction of the electric field between the source and drain), the molecular orientation of BuPTCD favors the charge carrier mobility (Figure S4). Furthermore, the chain-on organization of BuPTCD can enhance the BuPTCD/Au contact area, reducing contact resistance and consequently leading to higher *I*_DS_ values in BuPTCD HGOFETs compared with those for PhPTCD. Thus, the molecular organization may be a key factor contributing to the improved performance of BuPTCD HGOFETs. Finally, as μ_s_*C*_i_ is one of the main performance parameters, we utilized it for comparison with other electrolyte-gated OFETs (EGOFETs) fabricated with different organic materials. Table S1 shows the μ_s_*C*_i_ values of HGOFETs and water-gated OFETs (WGOFETs) fabricated with different organic materials, along with the average values obtained for HGOFETs made with BuPTCD and PhPTCD. It is observed that, in general, the μ_s_*C*_i_ values of HGOFETs fabricated with BuPTCD and PhPTCD are low but fall within the expected range for organic materials (Table S1).

**Table 1:** Summary of the Main Parameters and Figures of Merit Obtained from the Transfer Curves of BuPTCD and PhPTCD HGOFETs

perylene	BuPTCD			PhPTCD		
device	1	2	3	1	2	3
*I*_on_/*I*_off_	40	43	233	31	31	11
*g*_m_ (μ s^–1^)	0.78	0.65	0.57	0.37	0.26	0.23
SS (mV dec^–1^)	350.3	538.7	211.2	434	438.7	746
μ_s_*C*_i_ (nF V^–1^ s^–1^)	4.1	2.8	2.3	1.5	0.80	0.46
*V*_TH_ (V)	–0.7	–0.43	–0.4	–0.8	–0.84	–1.2
*I*_DS_/*I*_GS_	87	99	101	59	83	99

**Figure 7:**
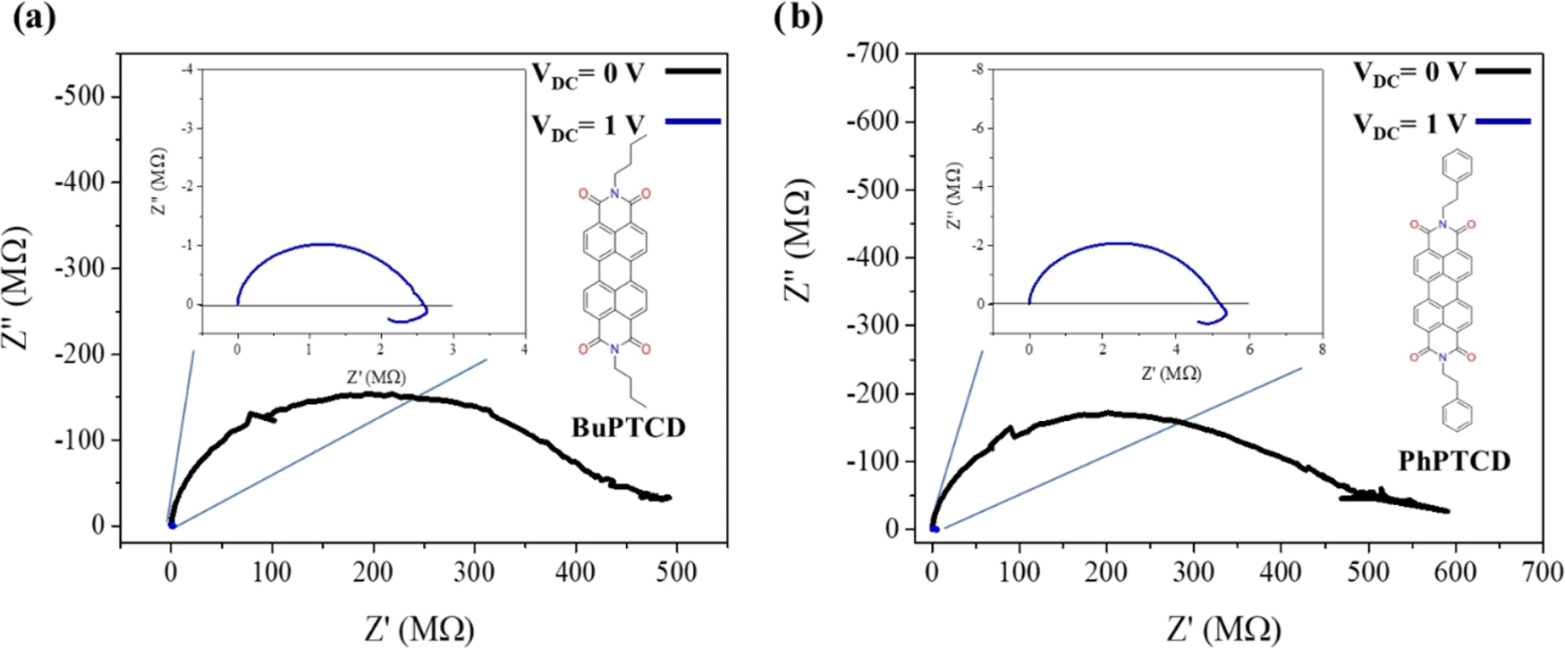
Nyquist plots in the dark for (a) BuPTCD and (b) PhPTCD under *V*_DC_ = 0 and *V*_DC_ = 1 V. Inset: zoom of the Nyquist plot for *V*_DC_ = 1 V.

Figure 6a,b displays the output curves (*I*_DS_*– V*_DS_) for BuPTCD and PhPTCD HGOFETs, respectively. These measurements were obtained with a scan rate of 10 mV s^–1^, where *V*_GS_ and *V*_DS_ varied from −0.2 to 1 V and from −0 to 1 V, respectively. The HGOFETs exhibited similar values for *I*_DS_; however, BuPTCD exhibited lower clockwise hysteresis (Figure 6a,b). In addition, both devices presented a non-ideal saturation regime, similar to observations in other studies involving OFETs.^48−50^ The existence of disorder or the presence of traps are factors that can result in a non-ideal saturation regime.^50^ Since the disorder is ruled out for both BuPTCD^46^ and PhPTCD^47^ films, as both exhibit molecular organization, it is likely that the observed effect is due to the presence of traps at the interface semiconductor/contact, as discussed in Figure 7a,b. Finally, it is interesting to note that the hysteresis of the output curves is in the opposite direction from that of the transfer curves. These results are similar to those obtained for water-GOFETs^18^ and transistor-type organic memory devices.^51^ However, the origin of this phenomenon is not yet clear.

Figure 7a,b illustrates Nyquist plots based upon impedance spectroscopy data for the BuPTCD and PhPTCD films in the dark under bias voltage, *V*_DC_ of 0 and 1 V with *V*_AC_ = 100 mV. It is observed that for *V*_DC_ = 1 V (same voltage applied between the source and drain in the transistor), there is the closure of the semicircle at low frequencies in both films. This can be attributed to a negative capacitance that is associated with the presence of traps. These traps may manifest at either the semiconductor/contact interface^52,53^ or grain boundaries.^54^ The possibility of grain boundaries can be ruled out because the XRD results show that the BuPTCD film is crystalline, but the PhPTCD film is amorphous (Figure S5). Regarding the interface, the deposition of the organic molecule active layer on bare Au promotes the presence of traps at the interface,^55,56^ as previously observed for a perylene derivative,^57^ and this could be the source of the negative capacitance and of a non-ideal saturation regime (Figure 6). Finally, it is important to mention that XRD reveals that crystallinity is not a fundamental parameter for the functioning of the transistors, as the PDI film also shows a crystalline phase (Figure S5).

**Figure 8:**
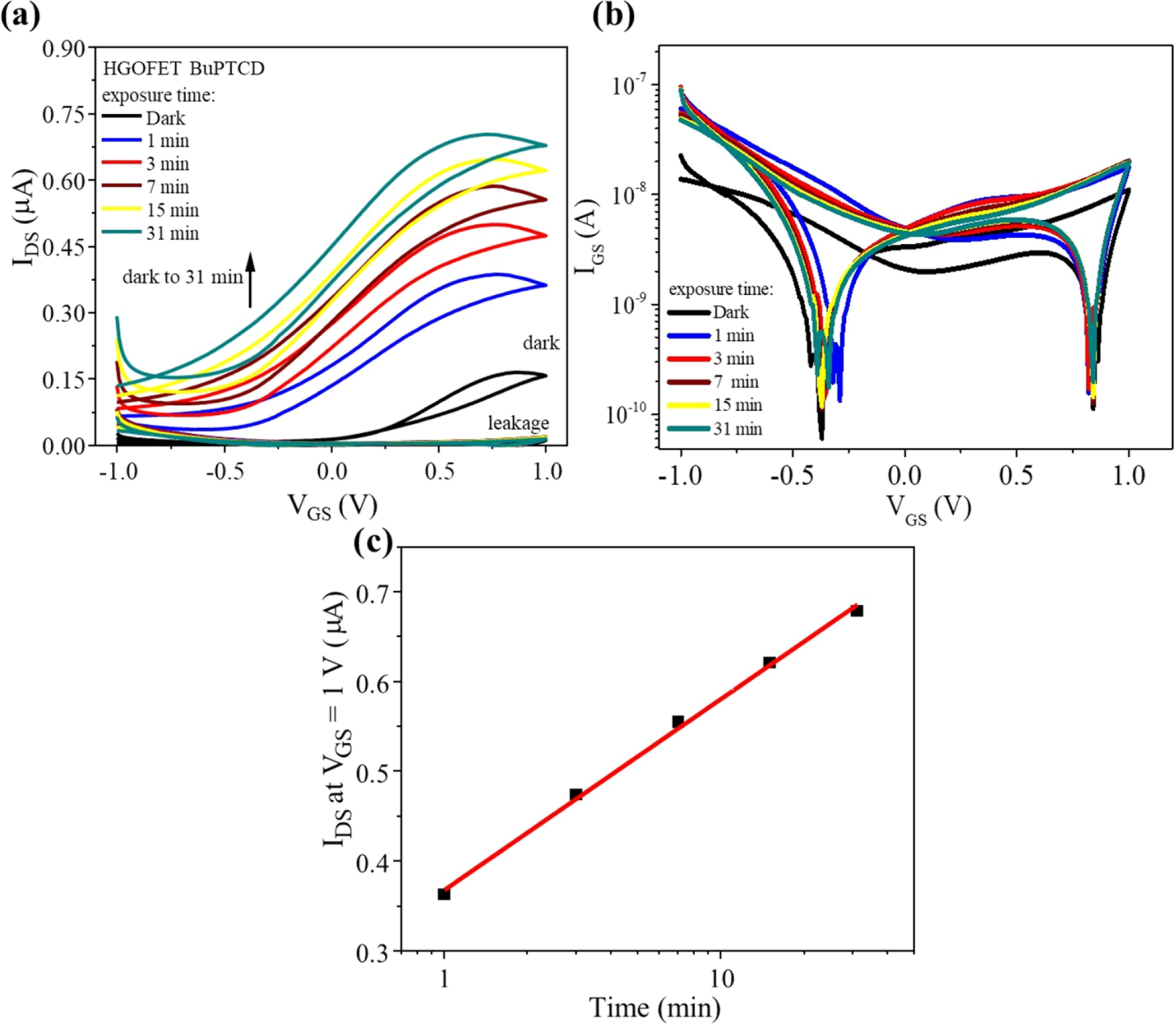
(a) Transfer curves at *V*_DS_ = 0.2 V for the BuPTCD HGOFET alongside leakage currents in a linear scale, (b) leakage currents for the BuPTCD HGOFET at *V*_DS_ = 0.2 V in a semilogarithmic scale, and (c) *I*_DS_ at *V*_DS_ = 0.2 V and *V*_GS_ = 1 V under UV radiation for different exposure times (1, 3, 7, 15, and 31 min).

The BuPTCD and PhPTCD HGOFETs were assessed as potential targets for UV light. UV light detectors using wavelengths tailored toward the perylene absorption peaks have already been demonstrated;^58−60^ this work focused upon excitation of the devices in the wavelengths where perylene is known to absorb weakly (Figure S1), but it is the range where UV radiation has the highest irradiance (near 400 nm).^61^ Transfer measurements were obtained using a double sweep transfer (*I*_DS_*–V*_GS_) along with the leakage current (*I*_GS_–*V*_GS_) at a scanning rate of 10 mV s^–1^ and *V*_DS_ = 0.2 V, both in the dark and under UV radiation with λ = 390 nm and *P*_d_ = 0.5 mW cm^–2^ (Figures 8 and S6). The transfer was carried out using *V*_DS_ = 0.2 V because in this condition, it achieved a better *I*_on_/*I*_off_ ratio and a more significant photocurrent. During the measurements, the HGOFETs were continuously irradiated, and transfer measurements were collected at various exposure times (1, 3, 7, 15, and 31 min). Since the results for the BuPTCD and PhPTCD HGOFETs were similar, only the results for BuPTCD are presented in Figure 8, and those for PhPTCD are available in the SI (Figure S6). Figure 8a demonstrates a significant increase in the measured *I*_DS_ over time and a decrease in the *I*_on_/*I*_off_ ratio. The reduction in the *I*_on_/*I*_off_ ratio is attributed to the increased number of charge carriers in the active layer generated due to light absorption, which consequently hinders transistor switching behavior, as observed in other works.^62−64^Figure 8b demonstrates a slight increase in *I*_GS_, reaching around 10 nA after 1 min under UV light, followed by stable readings over subsequent time intervals (3, 7, 15, and 31 min). Figure 8c shows that *I*_DS_ at *V*_GS_ = 1 V linearly increases with the logarithm of time, demonstrating that HGOFETs based on BuPTCD and PhPTCD possess photoconductive properties and can measure UV light as a function of exposure time. Transfer curves of HGOFETs were also measured under different UV power intensities, and both devices exhibited a linear increase in *I*_DS_ with UV power densities (Figure S7), similar to the behavior observed as a function of exposure time (Figure 8c). This linear relationship suggests that the devices maintain consistent sensitivity across the range of UV intensities tested, highlighting their potential for real monitoring of UV radiation with excellent photoconductive properties.

## 4 Conclusions

New concepts of sustainable devices are required to meet the future demands of the ICT industry. The research has focused on the use of perylene derivatives as the active layer for transistors, demonstrating the impact of their chemical structure on the electrical properties of the active layer. To achieve this, we are developing transistors that are sustainable and degrade into non-hazardous products at the end of their lifecycle. AC measurements revealed that the films of BuPTCD and PhPTCD increased the number of carriers and improved their AC conductivity (at low frequencies) by approximately 10^2^ S m^–1^ at *V*_DS_ = 1 V. Conversely, the films of PDI and PDI-ONaph showed fewer changes. This phenomenon was ascribed to the larger intermolecular distance (weaker π-stacking interaction) in the PDI and PDI-ONaph films due to the second chain containing four carbon atoms at the imide position, which compromised the fabrication of HGOFETs. The HGOFETs of BuPTCD and PhPTCD were successfully fabricated, and their merits were extracted from the transfer curves. The BuPTCD HGOFETs exhibited an average value of μ_s_*C*_i_ greater than that of PhPTCD, attributed to the chain-on molecular organization of BuPTCD that favors charge carrier movement. UV light detection results were obtained from the HGOFET, and good performance was observed, despite the low absorption of light in perylene within the UV region. Finally, it is noteworthy that the integration of AC and optical measurements, coupled with the fabrication of HGOFETs, has proven to be important in analyzing chemical structures suitable for the manufacturing and optimization of transistors.

## Supporting Information Available

UV–vis absorption spectra (for films and solutions) and emission spectra (for films) of perylene derivatives; transfer curves for BuPTCD and PhPTCD HGOFETs; schematic illustrations of molecular orientation of BuPTCD and PhPTCD in the film; comparison table of μ_s_*C*_i_ values for BuPTCD and PhPTCD HGOFETs with other organic materials; XRD diffractogram of perylene derivative films; transfer curves for the PhPTCD HGOFET under UV radiation at different exposure times (1, 3, 7, 15, and 31 min); and *I*_DS_ for BuPTCD and PhPTCD HGOFETs under UV radiation at different power densities (PDF)
