# Supplementary material for: Influence of the Chemical Structure of Perylene Derivatives on the Performance of Honey-Gated Organic Field-Effect Transistors (HGOFETs) and Their Application in UV Light Detection
Source: ACS Appl Electron Mater. 2024 Nov 22;6(12):9142–53. doi: 10.1021/acsaelm.4c01773 (PMC11673095; doi:10.1021/acsaelm.4c01773)
Supplement: Supplementary file 1 — el4c01773_si_001.pdf [file el4c01773_si_001.pdf]

Supporting Information

Influence of Perylene Derivatives Chemical Structure on the Performance of Honey-Gate Organic Field-Effect Transistors (HGOFETs) and Their Application in UV Light Detection

Jose Diego Fernandes[1,3], Douglas Henrique Vieira[1], Theodoros Serghiou[3], Carlos J. Rivas[2], Carlos J. L. Constantino[1], Liliana B. Jimenez[2], Neri Alves[1], Jeff Kettle[3*]

[1]Department of Physics, School of Technology and Applied Sciences, São Paulo State University (UNESP), Presidente Prudente, SP, Brazil.

[2]Departamento de Química Orgánica, Facultad de Ciencias Químicas, Universidad Nacional de Córdoba. INFIQC, Instituto de Investigaciones en Físicoquímica de Córdoba (CONICET-UNC). Córdoba, X5000HUA, Argentina.

[3]James Watt School of Engineering, University of Glasgow, G12 8QQ Glasgow, Scotland, U.K.

*Corresponding Author: Jeff.Kettle@glasgow.ac.uk

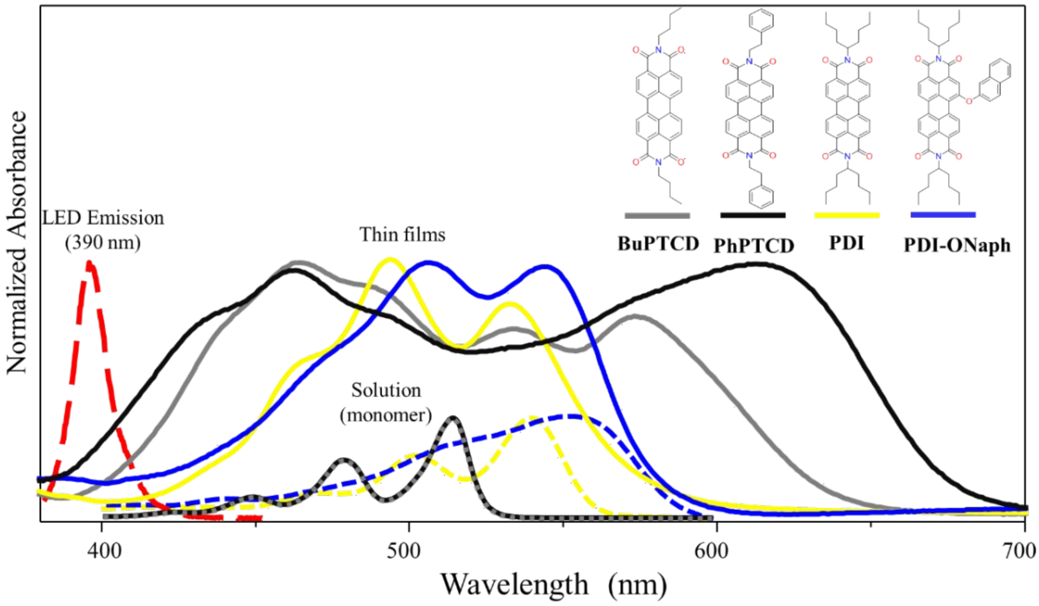

Figure S1: normalized UV-Vis absorption spectrum for the films and solutions in DCM/TFA at a 9:1 (v/v) of BuPTCD (grey line), PhPTCD (black line), PDI (yellow line), and PDI-ONaph (blue line), respectively, alongside the LED emission spectrum (red dashed line). All films were heat treated at 100ºC for 2 h.

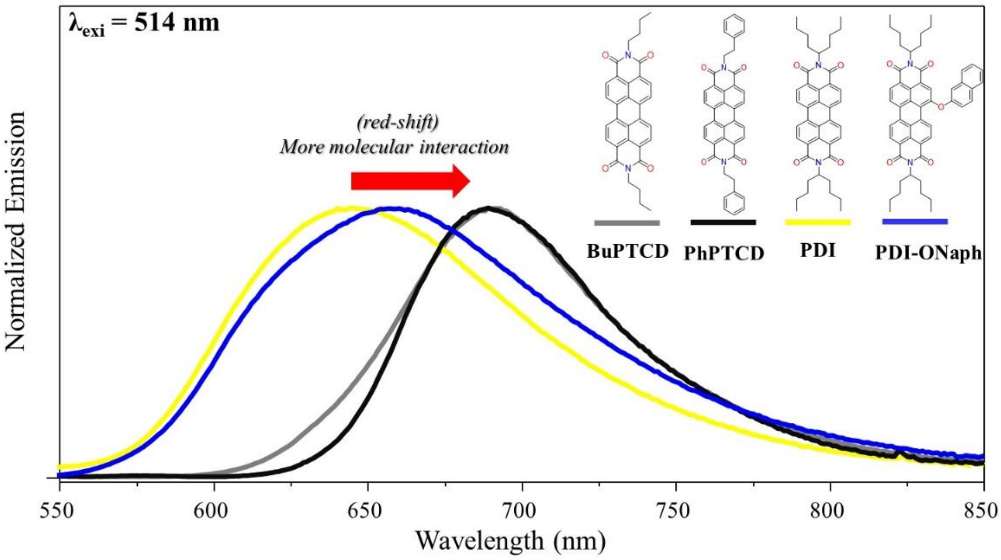

Figure S2: normalized emission spectrum of films BuPTCD (grey line), PhPTCD (black line), PDI (yellow line), and PDI-ONaph (blue line). All films were heat treated at 100ºC for 2 h.

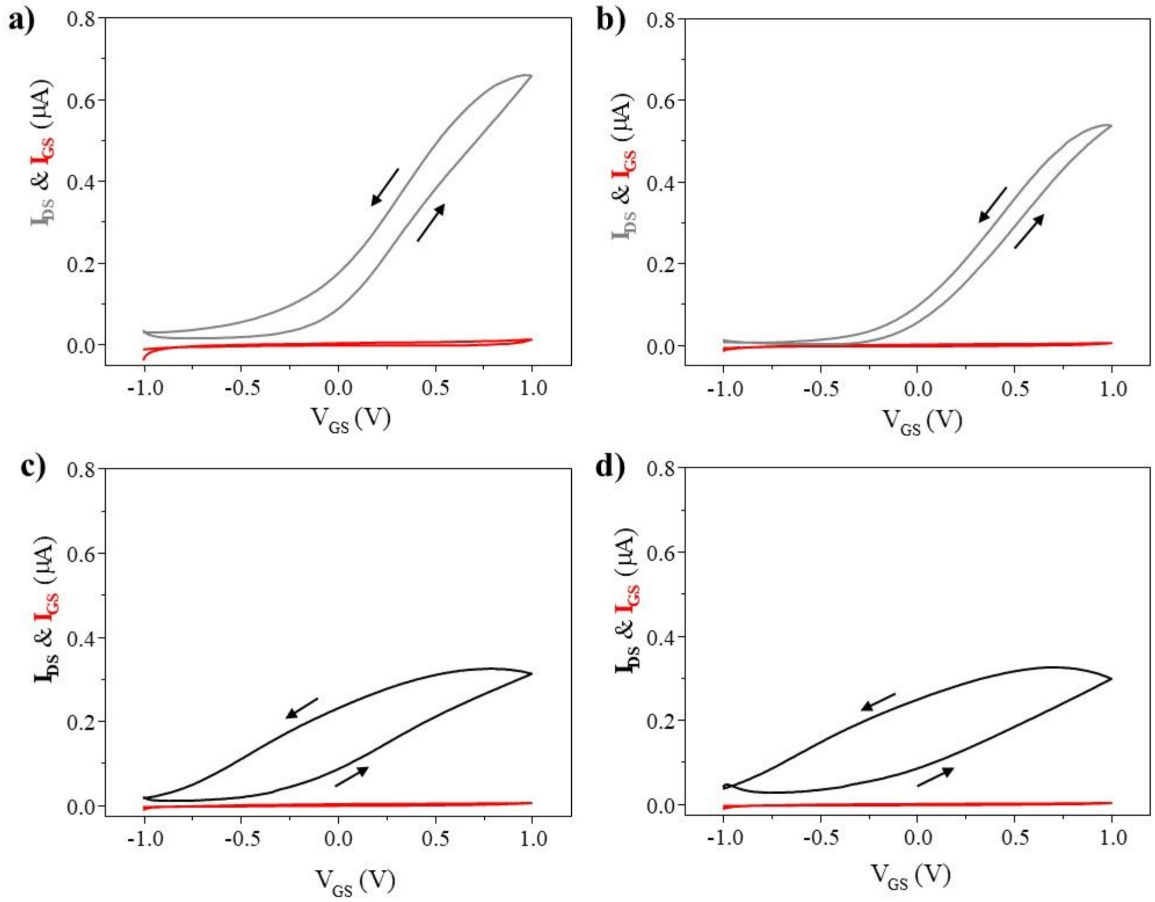

Figure S3: transfer curves of HGOFETs for samples 2 and 3 of a) and b) BuPTCD, and c) and d) PhPTCD, measured under V_{DS} = 1 V. The red lines represent the corresponding leakage currents.

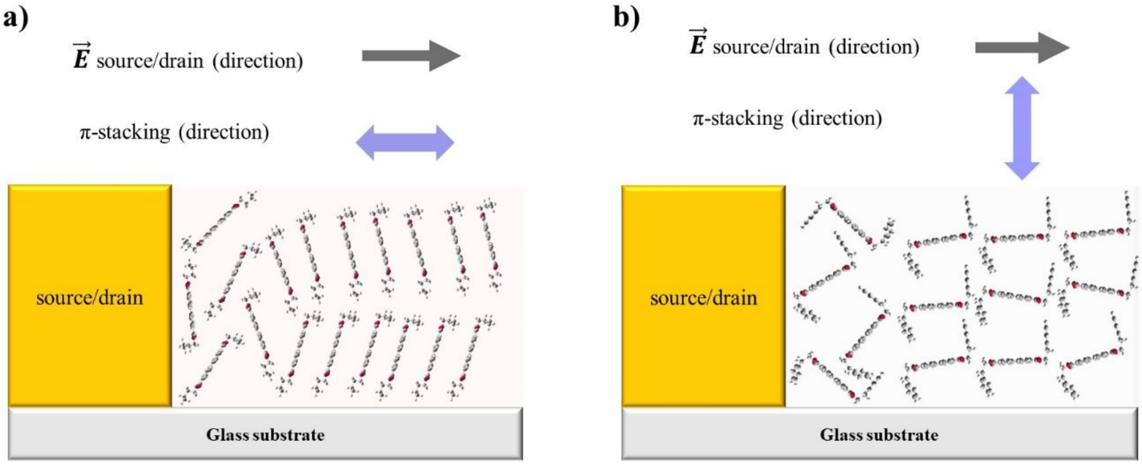

Figure S4: Schematic illustrations of the molecular orientation in films of a) BuPTCD (chain-on) and b) PhPTCD (face-on), highlighting the region near the Au contact/perylene interface. The electric field ($\vec{E}$) and π-stacking interaction direction are indicated by the arrows above.

Table 1SI: The comparison of the $\mu_s C_i$ values of HGOFETs and Water-GOFETs (WGOFETs) fabricated with different organic materials, along with the best values obtained for HGOFETs made with BuPTCD and PhPTCD.

Devices	$\mu_s C_i$ (nF $V^{-1}s^{-1}$)	References
$PBTTT_{WGOFET}$	50	[1]
$PNDIT2_{HGOFET}$	23	[2]
$PDPPDTT_{WGOFET}$	20	[1]
$PIDTBT_{WGOFET}$	13	[1]
$PCBM_{WGOFET}$	7.0	[3]
$P3HT_{WGOFET}$	6.0	[4]
$BuPTCD_{HGOFET}$	4.1	(average, this work)
$CuPc_{WGOFET}$	4.0	[5]
$P3HT_{HGOFET}$	3.5	[2]
$PNDISVS_{WGOFET}$	2.5	[3]
$PhPTCD_{HGOFET}$	1.5	(average, this work)
$PNDIT2_{WGOFET}$	0.3	[3]

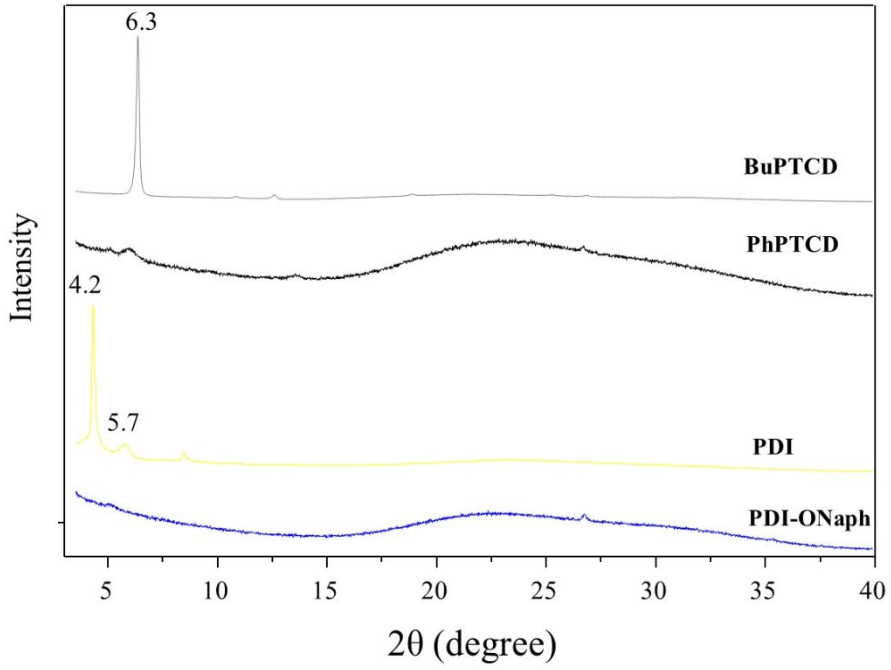

Figure S5: XRD diffractogram for BuPTCD (grey line), PhPTCD (black line), PDI (yellow line) and PDI-ONaph (blue line) films. All films were heat treated at 100ºC for 2 h.

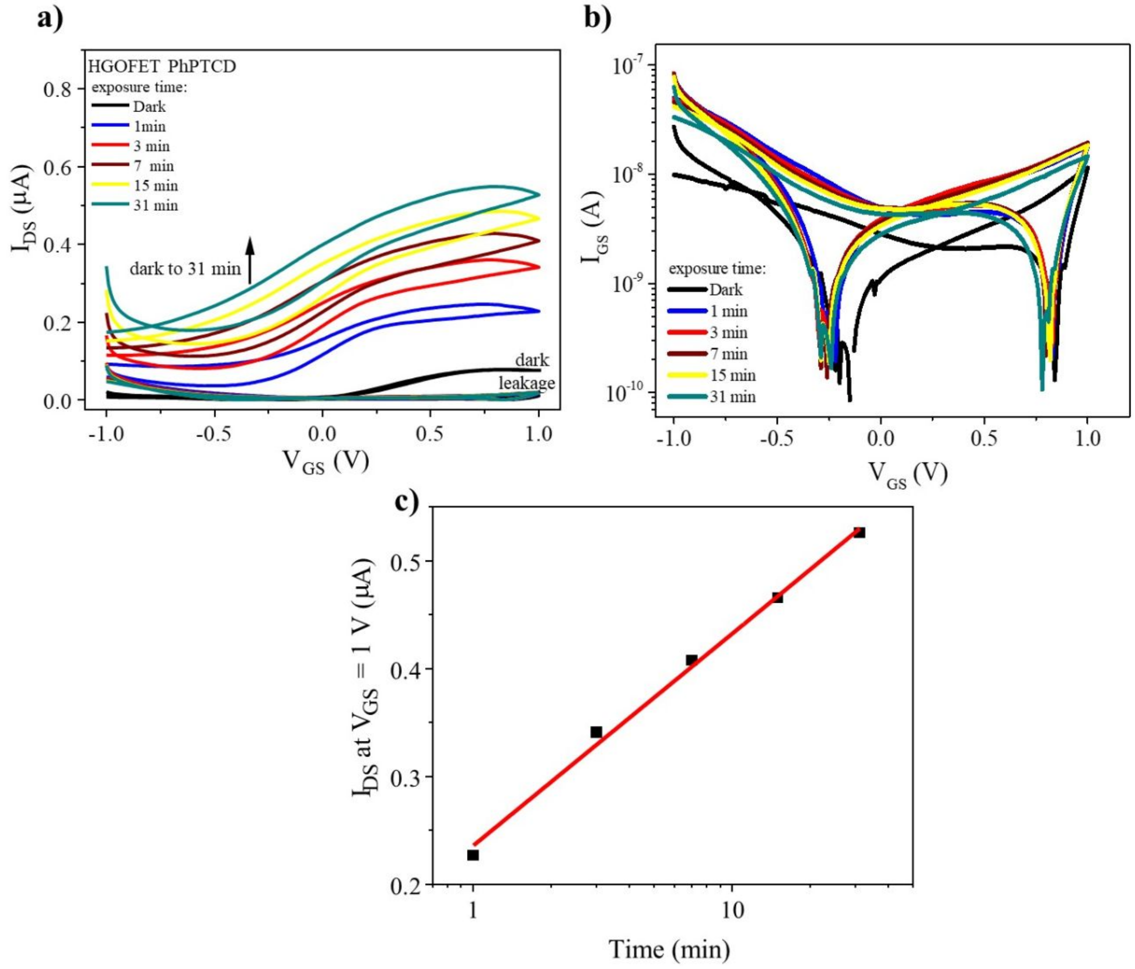

Figure S6: a) transfer curves at V_{DS} = 0.2 V for the PhPTCD HGOFET alongside leakage currents in a linear scale; b) leakage currents for the PhPTCD HGOFET at V_{DS} = 0.2 V in a semi-logarithmic scale and c) I_{DS} at V_{DS} = 0.2 V and V_{GS} = 1 V under UV radiation for different exposure times (1, 3, 7, 15 and 31 minutes).

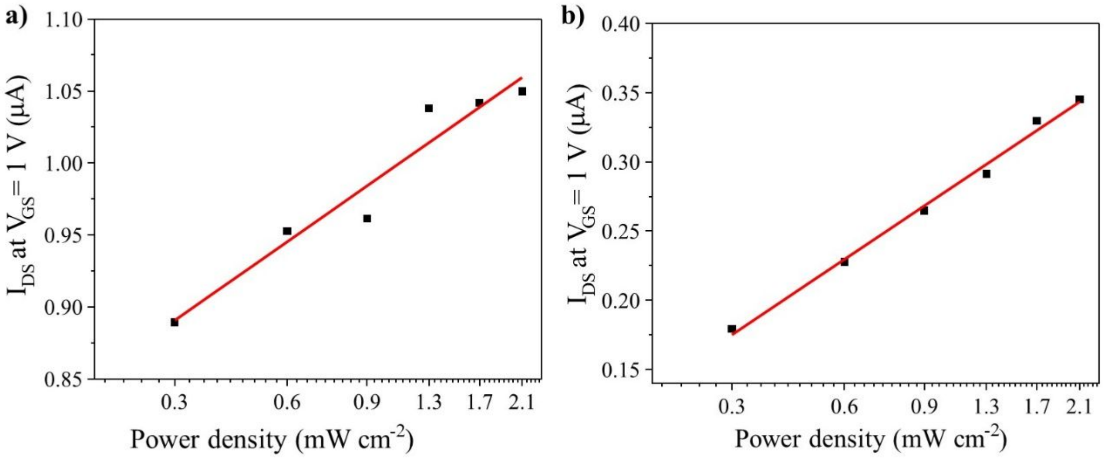

Figure S7: I_{DS} at V_{DS} = 0.2 V and V_{GS} = 1 V under UV radiation for different power densities. a) BuPTCD and b) PhPTCD HGOFETs.

References:

(1) Doumbia, A.; Tong, J.; Wilson, R. J.; Turner, M. L. Investigation of the Performance of Donor–Acceptor Conjugated Polymers in Electrolyte-Gated Organic Field-Effect Transistors. *Adv. Electron. Mater.* **2021**, *7* (9). https://doi.org/10.1002/aelm.202100071.

(2) Sharova, A. S.; Caironi, M. Sweet Electronics: Honey-Gated Complementary Organic Transistors and Circuits Operating in Air. *Adv. Mater.* **2021**, *33* (40). https://doi.org/10.1002/adma.202103183.

(3) Porrazzo, R.; Luzio, A.; Bellani, S.; Bonacchini, G. E.; Noh, Y.-Y.; Kim, Y.-H.; Lanzani, G.; Antognazza, M. R.; Caironi, M. Water-Gated n-Type Organic Field-Effect Transistors for Complementary Integrated Circuits Operating in an Aqueous Environment. *ACS Omega* **2017**, *2* (1), 1–10. https://doi.org/10.1021/acsomega.6b00256.

(4) Porrazzo, R.; Bellani, S.; Luzio, A.; Bertarelli, C.; Lanzani, G.; Caironi, M.; Antognazza, M. R. Field-Effect and Capacitive Properties of Water-Gated Transistors Based on Polythiophene Derivatives. *APL Mater.* **2015**, *3* (1). https://doi.org/10.1063/1.4900888.

(5) de Oliveira, R. F.; Merces, L.; Vello, T. P.; Bof Bufon, C. C. Water-Gated Phthalocyanine Transistors: Operation and Transduction of the Peptide–Enzyme Interaction. *Org. Electron.* **2016**, *31*, 217–226. https://doi.org/10.1016/j.orgel.2016.01.041.
